# Exosome-derived miR-142-5p remodels lymphatic vessels and induces IDO to promote immune privilege in the tumour microenvironment

**DOI:** 10.1038/s41418-020-00618-6

**Published:** 2020-09-14

**Authors:** Chenfei Zhou, Yanmei Zhang, Ruiming Yan, Lei Huang, Andrew L. Mellor, Yang Yang, Xiaojing Chen, Wenfei Wei, Xiangguang Wu, Lan Yu, Luojiao Liang, Dan Zhang, Sha Wu, Wei Wang

**Affiliations:** 1 Department of Obstetrics and Gynecology, The First Affiliated Hospital of Guangzhou Medical University, Guangzhou, 510120 China; 2 Department of Immunology/Guangdong Provincial Key Laboratory of Proteomics, School of Basic Medical Sciences, Southern Medical University, Guangzhou, 510515 China; 3 Institute of Cellular Medicine, Faculty of Medical Sciences, Framlington Place, Newcastle University, Newcastle-Upon-Tyne, NE2 4HH UK; 4 Huiqiao Medical Center, Nanfang Hospital, Southern Medical University, Guangzhou, 510515 China

**Keywords:** Cancer microenvironment, Oncogenes

## Abstract

Clinical response to immunotherapy is closely associated with the immunosuppressive tumour microenvironment (TME), and influenced by the dynamic interaction between tumour cells and lymphatic endothelial cells (LECs). Here, we show that high levels of miR-142-5p positively correlate with indoleamine 2,3-dioxygenase (IDO) expression in tumour-associated lymphatic vessels in advanced cervical squamous cell carcinoma (CSCC). The miR-142-5p is transferred by CSCC-secreted exosomes into LECs to exhaust CD8^+^ T cells via the up-regulation of lymphatic IDO expression, which was abrogated by an IDO inhibitor. Mechanistically, miR-142-5p directly down-regulates lymphatic AT-rich interactive domain-containing protein 2 (ARID2) expression, inhibits DNA methyltransferase 1 (DNMT1) recruitment to interferon (IFN)-γ promoter, and enhances IFN-γ transcription by suppressing promoter methylation, thereby leading to elevated IDO activity. Furthermore, increased serum exosomal miR-142-5p levels and the consequent IDO activity positively correlate with CSCC progression. In conclusion, exosomes secreted by CSCC cells deliver miR-142-5p to LECs and induce IDO expression via ARID2–DNMT1–IFN-γ signalling to suppress and exhaust CD8^+^ T cells. Our study suggests that LECs act as an integral component of the immune checkpoint(s) in the TME and may serve as a potential new target for CSCC diagnosis and treatment.

## Introduction

Tumour progression has been considered as a consequence of the evolving crosstalk between different cell types within the tumour and its stroma [1–3]. The remodelling of regional lymphatic vessels (LVs) conferred by tumour cells is associated with enhanced malignant progression and poor outcome in many solid tumours, including cervical squamous cell carcinoma (CSCC) [2]. Besides offering physical routes for metastatic spread [4, 5], tumour-associated LVs are thought to be involved in the modulation of adaptive immune responses [6]. These findings have focused research attention on tumour-associated lymphatic endothelial cells (LECs), one of many types of stromal cells, that help establish an immunosuppressive tumour microenvironment (TME), which promotes the evasion of anti-tumour immunity. A better understanding of the immunosuppressive mechanisms of tumour-associated LECs would thus facilitate the rational development of therapeutic strategies.

The recent discovery of miRNAs and their extracellular presence have highlighted the potential role of these regulatory molecules in mediating the cancer-stroma crosstalk [7]. In the extracellular space, miRNAs either bind to proteins or lipids [8] or serve as a major RNA component of exosomes [9]. Exosomes are small 30–150 nm membrane vesicles that are delivered into the extracellular environment by different cell types, including cancer cells [1]. These exosomes reflect the expression patterns of dysregulated miRNAs in cancer cells. Thus, cancer-secreted exosomes may deliver miRNAs to host recipient cells to modify gene expression on a genome-wide scale. The expression of the miR-142-5p that serves as a tumour suppressor has been reported to be down-regulated in gastric cancer [10], while it is up-regulated as a strong oncogene in cutaneous squamous cell carcinoma, colorectal cancer, and renal cell carcinoma [11–13]. However, there are no reports on the cancer-derived exosomal miR-142-5p that may shape the cancer immune landscape and create immune checkpoint(s) in the TME.

Indoleamine 2,3-dioxygenase (IDO) is an intercellular enzyme that converts the essential amino acid tryptophan into kynurenine through the IDO/kynurenine pathway [14]. IDO activity is monitored by measuring kynurenine to tryptophan (K:T) ratio [15]. Elevated IDO activity correlates with a decrease in the population of tumour-infiltrating lymphocytes (TILs) in oesophageal and endometrial cancers [16, 17] and poor clinical outcomes in multiple types of cancers [18–20]. These clinical observations support the paradigm that IDO contributes to immune checkpoint development and promotes tumour progression by attenuating effector T cell responses. Although IDO may be expressed by tumour, stroma, and immune cells in the TME [21, 22], little is known about the molecular mechanism and function of IDO in tumour-associated LVs.

Here, we investigated whether cancer-secreted exosomal miR-142-5p modulates the tumour immune status of CSCC by inducing IDO expression and evaluated the molecular mechanism underlying miR-142-5p-mediated tumour progression to reveal the potential clinical applications of this miRNA in the diagnosis and therapy of CSCC.

## Materials and methods

### Cell lines

The human CSCC cell lines Siha, Caski, C33A, ME180, and MS751 were purchased from the American Type Culture Collection (ATCC) and cultured as per the guidelines. Human dermal lymphatic endothelial cells (HDLECs; IDO-negative) were obtained from ScienCell and cultured in endothelial cell medium (ECM; ScienCell) supplemented with 10% foetal bovine serum (FBS; Gibco) and endothelial growth medium supplements (ScienCell). In all related experiments, CSCC cell lines and HDLECs were used within 20 and 6 passages, respectively.

### Clinical samples

CSCC tissue microarrays were purchased from SHANGHAI OUTDO BIOTECH CO., LTD. Blood samples were obtained from voluntarily CSCC patients without preoperative radiotherapy or chemotherapy at the department of Gynaecological Oncology of the First Affiliated Hospital of Guangzhou Medical University (Guangzhou, China) or healthy donors between 2016 and 2017. The human study was approved by the World Medical Association Declaration of Helsinki and Institutional Research Ethics Committee at Ministry of Public Health of PR China. Written informed consent was obtained from all patients or healthy donors. All blood samples were centrifuged at 2500 × *g* for 10 min to extract serum. All samples were stored at –80 °C until further study.

### Exosome isolation, characterisation, and treatment

Exosomes were purified from CSCC-derived supernatant or the serum of CSCC patients by ultracentrifugation. Cell culture supernatants were collected after 48 h and centrifuged at 500 × *g* for 10 min at 4 °C and then at 10,000 × *g* for 30 min at 4 °C. The serum was diluted with an equal volume of phosphate-buffered saline (PBS) and centrifuged at 2000 × *g* for 30 min at 4 °C, followed by 12,000 × *g* for 45 min at 4 °C. The supernatants were then passed through 0.22 µm filters (Millipore) and ultra-centrifuged at 100,000 × *g* for 70 min at 4 °C. The exosome pellets were washed with PBS and subjected to a second ultracentrifugation step at 100,000 × *g* for 70 min at 4 °C and then resuspended in PBS. The exosomes were evaluated by Nanosight NS300 (Malvern). For transmission electron microscopy, exosomes were fixed with 2% glutaraldehyde and loaded on carbon-coated grids. The grids were then subjected to negative staining using phosphotungstic acid for 2 min and visualised by transmission electron microscopy (Hitachi H-7500). For the exosome uptake study, PKH67 membrane dye (Sigma) was added to PBS at 1 µM concentration and incubated with exosomes for 20 min before washing. The excess dye was removed by an additional washing step, and the labelled exosomes were resuspended and used to treat HDLECs. After a 48-h incubation, HDLECs were labelled with phalloidin for confocal imaging. For treatment of cells and animals, 2 µg exosomes, as measured using a bicinchoninic acid (BCA) protein assay kit (Beyotime), were added to 1 × 10^5^ recipient cells for 48 h.

### Real-time quantitative polymerase chain reaction (RT-qPCR)

The RNA was extracted from cells and exosomes using the miRNeasy Mini Kit (Qiagen) according to the manufacturer’s instructions. Reverse transcription was performed using the Mir-X™ miRNA First-Strand Synthesis Kit (TaKaRa) for miRNAs or PrimeScript™ RT Master Mix (TaKaRa) for general genes. RT-qPCR was conducted using SYBR Premix Ex Taq™ (TaKaRa) on an Applied Biosystems 7500 Fast Real-Time PCR System (Applied Biosystems). Specific primer sets for miR-142-5p and U6 were obtained from RiboBio Inc. The expression of miRNAs and mRNAs was normalised to *U6* and *GAPDH*, respectively. The primer sequences are shown in Supplementary Table S1.

### Western blot analysis

Cell and exosome lysates were prepared in radioimmunoprecipitation assay (RIPA) buffer (FUDE BioTech) and quantified using Bradford protein assay (Beyotime). The lysates were subjected to sodium dodecyl sulphate polyacrylamide gel electrophoresis (SDS-PAGE) and the separated protein bands were transferred onto polyvinylidene fluoride membranes (Millipore). The membranes were incubated with primary antibodies for overnight at 4 °C, and then probed with a specific horseradish peroxidase (HRP)-conjugated anti-rabbit or anti-mouse immunoglobulin-G antibody (ab6721 or ab6728, Abcam). The chemiluminescence signal was detected using the FD8020 FDbio-Dura ECL Kit (FUDE BioTech). The primary antibodies used in this study are shown in Supplementary Table S2.

### Fluorescence in situ hybridisation (FISH) and immunohistochemistry (IHC)

FISH was performed on CSCC tissue microarrays using a fluorescence ISH kit (Boster BioTech) and the miR-142-5p synthetic oligonucleotide probes (Exiqon), according to the manufacturer’s protocol. In brief, the slides were incubated with 3% hydrogen peroxide (H_2_O_2_), digested with pepsin for 2 min at 37 °C, and fixed with 1% paraformaldehyde (PFA) in diethyl pyrocarbonate (DEPC) for 5 min. The slides were then pre-hybridised in a hybridisation buffer at 42 °C with miR-142-5p or U6 probes and incubated with fluorescent streptavidin-biotin complex (f-SABC) and horseradish peroxidase polymer. For IHC, sections were immersed in 3% H_2_O_2_ to block endogenous peroxidase activity and incubated with primary antibodies, overnight at 4 °C. A horseradish peroxidase-conjugated anti-rabbit secondary antibody (ZSGB BioTech) was subsequently applied for 1 h at room temperature. The expression of D240 (M361929-2, DAKO), IDO (86630, CST), and CD8 (ab93278, Abcam) was visualised using 3,3′-diaminobenzidine (DAB) following counterstaining with haematoxylin (ZSGB BioTech). FISH- and IHC-stained tissue sections were reviewed and scored separately by two independent pathologists. For semi-quantitative evaluation of IDO and miR-142-5p expression in tissue sections, the German Immuno-Reactive Score was applied as previously described [23].

### Multiplexed immunofluorescence staining

Multiplexed immunofluorescence staining was performed by OPAL 4-colour fluorescence IHC Kit (Perkin Elmer) according to manufacturer’s protocol. The following primary antibodies were used: D240 (M361929-2, DAKO) and IDO (86630, CST). After deparaffinization, steps were repeated for each primary antibody and sections were microwaved in an antigen retrieval buffer for 45 s at 100 °C. The slides were washed and blocked for 10 min at room temperature, and then incubated with a primary antibody. Next, slides were incubated with SuperPicture Polymer Detection Kit-HRP-broad spectrum (Life Technologies), and subsequently treated with Opal fluorochromes (Opal520 and Opal570) diluted 1:150 in an amplification buffer (all provided by the OPAL 4-colour fluorescence IHC kit) for 10 min at room temperature. Finally, a microwave treatment with AR6 buffer was performed. The slides were then incubated with a 4′,6-diamidino-2-phenylindole (DAPI) working solution (provided by the OPAL 4-colour fluorescence IHC kit) for 5 min at room temperature and then mounted under coverslips with ProLong Diamond Antifade Mountant (Life Technologies).

### Stable transfection with lentiviral vector

Lenti-GFP containing an miR-142-5p overexpression sequence and its negative control RNA (miR-NC) were all purchased from GeneChem Inc. Siha and Caski cells were transfected with lenti-GFP/miR-142-5p or lenti-GFP/miR-NC. Polyclonal cells with green fluorescent protein signals were purified for further experiments using a fluorescence-activated cell sorting flow cytometer.

### Transient transfection with oligonucleotides and plasmids

The miR-142-5p mimics and their negative control (miR-NC) were designed and cloned by RiboBio Inc. The ARID2 coding sequence (without 3’-UTR) was cloned into the pCDNA3.1(+)-Vector (Invitrogen). The empty vector was used as a blank control. SiARID2 and its negative control (siRNA) were designed and synthesised by GenePharma Inc. Lipofectamine 2000 Reagent (Invitrogen) was then used to transfect miR-142-5p mimics or inhibitors, siARID2, and pCDNA3.1(+)-ARID2 according to the manufacturer’s protocol. For RNA extraction, western blot and in vitro assays, cells were used 48 h after transfection. The sequences of siARID2 and siRNA are shown in Supplementary Table S1.

### In vivo xenograft model

Female B-NDG^®^ (NOD- *Prkdc*^*scid*^*IL2rg*^*tm1*^/Bcgen) mice (5-weeks-old) were purchased from Beijing Biocytogen Co., Ltd. Siha or Caski cells (1 × 10^5^) mixed with conditioned HDLECs (1 × 10^5^) treated with miR-142-5p mimics or exosomes from Siha/miR-142-5p or Caski/miR-142-5p were randomly injected into the right flank of each mouse (*n* = 6 per group). The tumours were harvested for analysis after 15 days. All animal studies were approved by the Institutional Animal Research Ethics Committee of Guangzhou Medical University.

### Human-activated CD8^+^ T cell preparation

Peripheral blood mononuclear cells (PBMCs) were isolated from healthy donors using Ficoll-Paque Plus (GE Healthcare). CD8^+^ T cells were isolated from PBMCs by magnetic bead purification using a human CD8^+^ T cell enrichment kit (STEMCELL Technologies). The purity (*>* 95%) was evaluated by flow cytometry using an eF450-labelled antibody against CD8 (48–0087–42, Thermo Fisher). CD8^+^ T cells were cultured in complete Roswell Park Memorial Institute (RPMI)-1640 medium. Then, CD8^+^ T cells (1 × 10^5^) were activated by stimulation with plate-bound anti-CD3 (OKT3, Thermo Fisher) at 2.5 µg/mL and anti-CD28 (10F3, Thermo Fisher) at 2 µg/mL in vitro.

### In vitro CD8^+^ T cell immune suppression

HDLECs (1 × 10^5^) treated as indicated with or without 100 µM BMS-986205 (HY-101560, MCE) were co-cultured with activated CD8^+^ T cells (1 × 10^5^) in the presence of a semipermeable transwell membrane (BD Biosciences). To access the cytotoxic activity of CD8^+^ T cells, the cells from 3-day co-cultures were subjected to surface or intracellular staining using the following anti-human antibodies or controls: fluorescein isothiocyanate (FITC)-conjugated anti-CD69 (11–0699–42, Thermo Fisher), phycoerythrin (PE)-conjugated anti-interferon (IFN)-γ (12–7319–42, Thermo Fisher), allophycocyanin (APC)-conjugated anti-programmed cell death protein-1 (PD-1; 17–2799–42, Thermo Fisher), or a fluorochrome-conjugated control IgG isotype. For analysis of CD8^+^ T cell apoptosis, the proportion of apoptotic CD8^+^ T cells was examined using an annexin V-APC apoptosis detection kit (KGF004, KeyGEN Biotech) after 7 days of co-cultures. Flow cytometry was performed using FACS LSRFortessa (BD Biosciences), and data were analysed with FlowJo VX software.

### Measurements of tryptophan and kynurenine

Supernatant or serum levels of tryptophan and kynurenine were measured using high-performance liquid chromatography (HPLC; UltiMate 3000, Thermo Fisher), as previously described with minor modifications [15]. For supernatants, 100 µL samples were diluted with a 1/10 volume of 150 mM NaAc (pH 4.0) and deproteinated with perchloric acid. For serum samples, 100 µL samples were diluted with an equal volume of 30 mM NaAc (pH 4.0) and deproteinated with 10% trichloric acid. In total, 50 µL of diluted samples were then loaded and analysed on a C18 column (Thermo Fisher). Tryptophan and kynurenine were detected on a UV channel at 280 and 360 nm, respectively. Samples were analysed using Dionex™ Chromeleon™ 7.0 software (Thermo Fisher) and quantified using external standards. At least one quality control sample was randomly inserted into every plate for reference. All assays were performed in triplicates, and each experiment was repeated thrice.

### Chromatin immunoprecipitation (ChIP) assay

Cells were crosslinked with 1% formaldehyde, quenched in a glycine solution, and subjected to CHIP assay using an enzymatic chromatin IP kit (#9003, CST) according to the manufacturer’s protocol. Anti-AT-rich interactive domain-containing protein 2 (ARID2) (orb386710, Biobyt), anti-DNA methyltransferase 1 (DNMT1) (61467, active motif), and normal IgG (Millipore) antibodies were used for immunoprecipitation. ChIP-enriched DNA samples were quantified by qPCR to determine ARID2- and DNMT1-binding sites in the IFN-γ promoter region. Data were shown as relative enrichment following normalisation to control IgG. The primer sequences used for ChIP-qPCR are shown in Supplementary Table S1.

### Methylation-specific polymerase chain reaction (MSP)

MSP was performed according to a previous study [24] using methylated primer pairs designed from the free online tool, MethPrimer (http://www.urogene.org/methprimer/index1.html). The methylated (M) and unmethylated (UM) primer sequences are shown in Supplementary Table S1.

### Other methods

Staining assessment, Luciferase activity assay, and ELISA assay were performed using the manufacturer’s protocol. See Supplementary Methods for more details.

### Statistical analysis

SPSS V.13.0 software was used for statistical analysis. Quantitative values in all experiments are expressed as the means ± standard deviation (SD). A two-tailed Student’s *t* test or one-way analysis of variance (ANOVA) was used for comparison between multiple groups. The Fisher or chi-square-test was applied for categorical variables. Correlation analysis was performed using the Spearman rank test. Differences were considered statistically significant at *P* < 0.05.

## Results

### miR-142-5p expression during CSCC progression correlates with lymphatic IDO expression and is negatively associated with the number of infiltrating CD8^+^ T cells

**Fig. 1:**
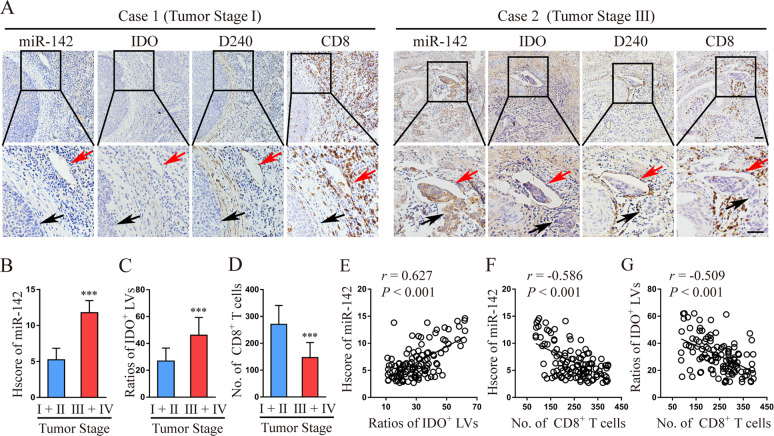
miR-142-5p expression correlates with lymphatic IDO expression and is negatively associated with the number of infiltrating CD8^+^ T cells during CSCC progression. **a** Representative micrographs of miR-142-5p, IDO, and D240 (lymphatic marker) staining in serial sections of CSCC specimens are shown. The LVs are indicated by red arrows. The tumour cells are indicated by black arrows. Scale bar: 50 µm. **b**–**d** ISH scores (Hscore) of miR-142-5p, ratio of IDO^+^ LVs to total LVs, and number of CD8^+^ T cells per field were analysed in serial sections from early-stage (FIGO 2018, stages I and II, *n* = 93) and advanced-stage (FIGO 2018, stages III and IV, *n* = 23) CSCC patients. **e**–**g** Correlation between miR-142-5p level, ratio of IDO^+^ LVs to total LVs, and number of CD8^+^ T cells was analysed. miR-142, miR-142-5p. Error bars represent the mean ± SD of three independent experiments. ***P < 0.001.

To evaluate the expression of miR-142-5p during CSCC progression, we analysed 116 human CSCC tissue microarrays using ISH. Compared with those in early stages (FIGO 2018, stages I and II, *n* = 93), miR-142-5p expression and the ratios of peritumoral IDO^+^ LVs indicated by both IDO- and D240-positive vessels were significantly higher in advanced stage CSCC patients (FIGO 2018, stages III and IV, *n* = 23), whereas the number of CD8^+^ T cells decreased during CSCC progression (Fig. 1a–d). Thus, miR-142-5p expression positively correlated with the ratio of peri-tumoural IDO^+^ LVs to total LVs (Fig. 1e). Further, miR-142-5p expression and the ratio of peri-tumoural IDO^+^ LVs to total LVs were negatively associated with the number of tumour-infiltrating CD8^+^ T cells (Fig. 1f, g). As shown in Fig. 1a, high lymphatic miR-142-5p and IDO expression co-localised with high miR-142-5p expression in the tumour, indicating that the tumour-derived miR-142-5p transferred to peri-tumoural LVs may up-regulate lymphatic IDO expression.

### miR-142-5p can be transferred to HDLECs via CSCC-secreted exosomes

**Fig. 2:**
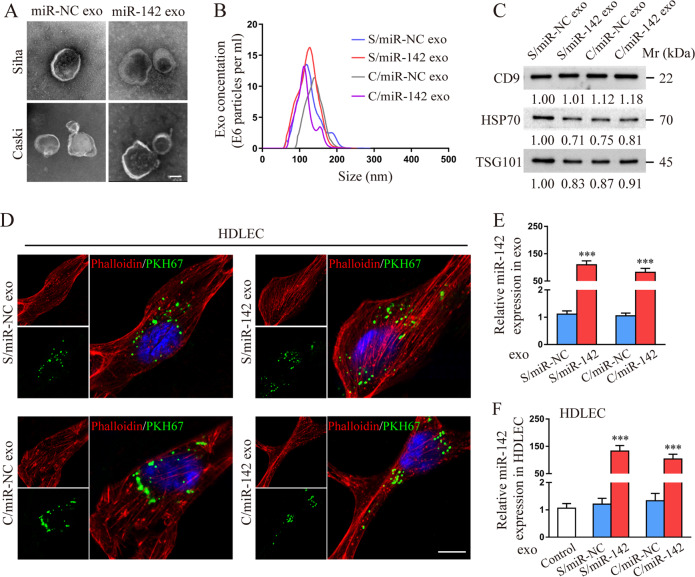
miR-142-5p can be transferred to human dermal lymphatic endothelial cells (HDLECs) via CSCC-secreted exosomes. **a** and **b** The morphology and size of exosomes secreted by Siha and Caski cells transfected with lentivectors overexpressing miR-142-5p or NC were confirmed by transmission electron microscopy and Nanosight particle tracking analysis. Scale bar, 50 nm. **c** Characteristic marker expression (CD9, HSP70, and TSG101) of exosomes was confirmed by western blotting. **d** Confocal imaging showed the delivery of PKH67-labelled exosomes (green) to phalloidin-labelled HDLECs (red). Scale bar, 25 µm. **e** miR-142-5p levels in indicated exosomes were detected by RT-qPCR. f miR-142-5p levels in HDLECs pre-treated with PBS or indicated exosomes for 24 h were detected by RT-qPCR. S/miR-NC exo Siha/miR-NC exosomes, S/miR-142 exo Siha/miR-142-5p exosomes, C/miR-NC exo Caski/miR-NC exosomes, C/miR-142 exo Caski/miR-142-5p exosomes. Error bars represent the mean ± SD of three independent experiments. ***P < 0.001.

Several recent studies have shown that cancer-secreted exosomal miRNAs regulate the crosstalk between malignant and stromal cells in the TME [25]. We, therefore, investigated whether miR-142-5p is involved in the crosstalk between CSCC cells and HDLECs through exosomes. Evaluation of all CSCC cell lines for of miR-142-5p expression showed that the level of miR-142-5p in Siha and Caski cells was similar to that observed in stage I tumour tissues but significantly lower than that reported in stage III tumour tissues (Supplementary Fig. S1a). Then, a lentiviral vector expressing miR-142-5p or negative control (NC) was successfully constructed and transfected into Siha and Caski cells (Supplementary Fig. S1b). Next, exosomes were isolated and purified from cell culture supernatants using standard ultracentrifugation. The cup-shaped structure, size, and number of isolated exosomes were identified by transmission electron microscopy and Nanosight particle tracking analysis (Fig. 2a, b). The isolated particles were confirmed to be exosomes through the characteristic expression of CD9, heat shock protein 70 (HSP70), and tumour susceptibility 101 (TSG101) (Fig. 2c). To evaluate the delivery of exosomes, the CSCC-secreted exosomes and HDLECs were labelled with PKH67 (green) and phalloidin (red), respectively. After 48 h incubation, confocal imaging was performed that revealed the appearance of green fluorescent spots in recipient HDLECs, indicating that the labelled exosomes secreted by CSCC cells were delivered to HDLECs (Fig. 2d).

RT-qPCR analysis showed a significant increase in the expression of miR-142-5p in the exosomes secreted by lenti-miR-142-5p-transfected Siha and Caski cells as compared with that in NC (Fig. 2e). To investigate whether the CSCC-secreted miR-142-5p is transferred to HDLECs via exosomes, we detected miR-142-5p level in HDLECs incubated with CSCC-secreted exosomes. The expression of miR-142-5p was higher in HDLECs incubated with exosomes with high miR-142-5p level than in HDLECs incubated with exosomes with low miR-142-5p (Fig. 2f), indicating the horizontal transfer of miRNA-142-5p from CSCC cells to HDLECs via exosomes.

### CSCC-secreted exosomal miR-142-5p up-regulates lymphatic IDO expression

**Fig. 3:**
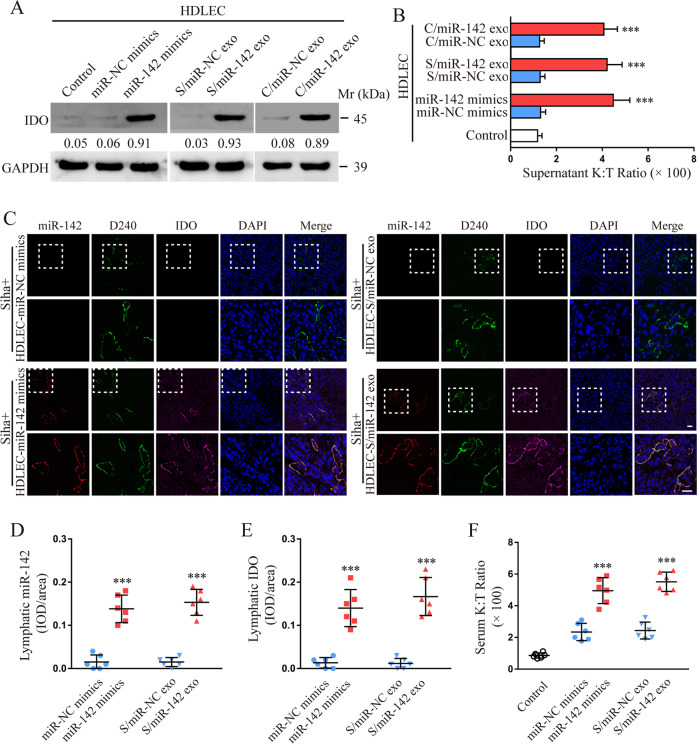
CSCC-secreted exosomal miR-142-5p up-regulates lymphatic IDO expression. **a** Protein level of IDO was detected in HDLECs pre-treated with miR-142-5p mimics or indicated exosomes by western blotting and compared with that in the control group. **b** The ratio of kynurenine to tryptophan (K:T) in the supernatant of HDLECs (1 × 10^5^ cells) after indicated treatment was calculated. **c** Representative micrographs of miR-142-5p, IDO, and D240 immunofluorescence staining in sections of xenograft tumours are shown. Scale bar: 50 µm. **d**–**f** The lymphatic expression of miR-142-5 and IDO and serum kynurenine to tryptophan (K:T) ratio in each group were calculated. S/miR-NC Siha/miR-NC, S/miR-142 Siha/miR-142-5p, C/miR-NC Caski/miR-NC, C/miR-142 Caski/miR-142-5p, exo exosomes. Error bars represent the mean ± SD of three independent experiments. ***P < 0.001.

To investigate the biological role of exosomal miR-142-5p in mediating lymphatic expression of IDO, we analysed the expression of IDO in HDLECs incubated with CSCC-secreted exosomes for 48 h by western blotting. IDO expression was significantly up-regulated in HDLECs treated with miR-142-5p mimics or exosomes containing miR-142-5p as compared with that in NC (Fig. 3a). As IDO enzyme degrades tryptophan and generates the tryptophan catabolite kynurenine [15], we assessed the concentrations of tryptophan and kynurenine in culture media using HPLC. An increase in kynurenine to tryptophan (K:T) ratio, indicative of elevated IDO activity, was observed in the supernatant of HDLECs treated with miR-142-5p mimics or exosomes containing miR-142-5p, but not in NC samples (Fig. 3b), consistent with the observation of IDO protein level.

To assess the role of exosomal miR-142-5p in the remodelling of the lymphatic phenotype in vivo, Siha cells mixed with conditioned HDLECs treated with exosomes from Siha/miR-142-5p or miR-142-5p mimics were subcutaneously injected into the right flanks of mice (*n* = 6 per group). After 15 days, the Siha cell group showed higher lymphatic expression of miR-142-5p and IDO (Fig. 3c–e) and serum K:T ratio (Fig. 3f) compared to the control group. Similar results were observed for the group exposed to Caski cells mixed with conditioned HDLECs treated with exosomes from Caski/miR-142-5p or miR-142-5p mimics (Supplementary Fig. S2a–d). In addition, HDLECs mixed with Siha/miR-NC, Siha/miR-142-5p, Caski/miR-NC, or Caski/miR-142-5p were used to construct the above xenograft model. The lymphatic expression of miR-142-5p and IDO (Supplementary Fig. S3a–c) and the serum K:T ratio (Supplementary Fig. S3d) were higher in the group of HDLECs mixed with Siha/miR-142-5p or Caski/miR-142-5p than in the group mixed with Siha/miR-NC or Caski/miR-NC. Taken together, CSCC-secreted exosomal miR-142-5p up-regulates the lymphatic expression of IDO in vitro and in vivo.

### CSCC-secreted exosomal miR-142-5p directly targets ARID2 in HDLECs

**Fig. 4:**
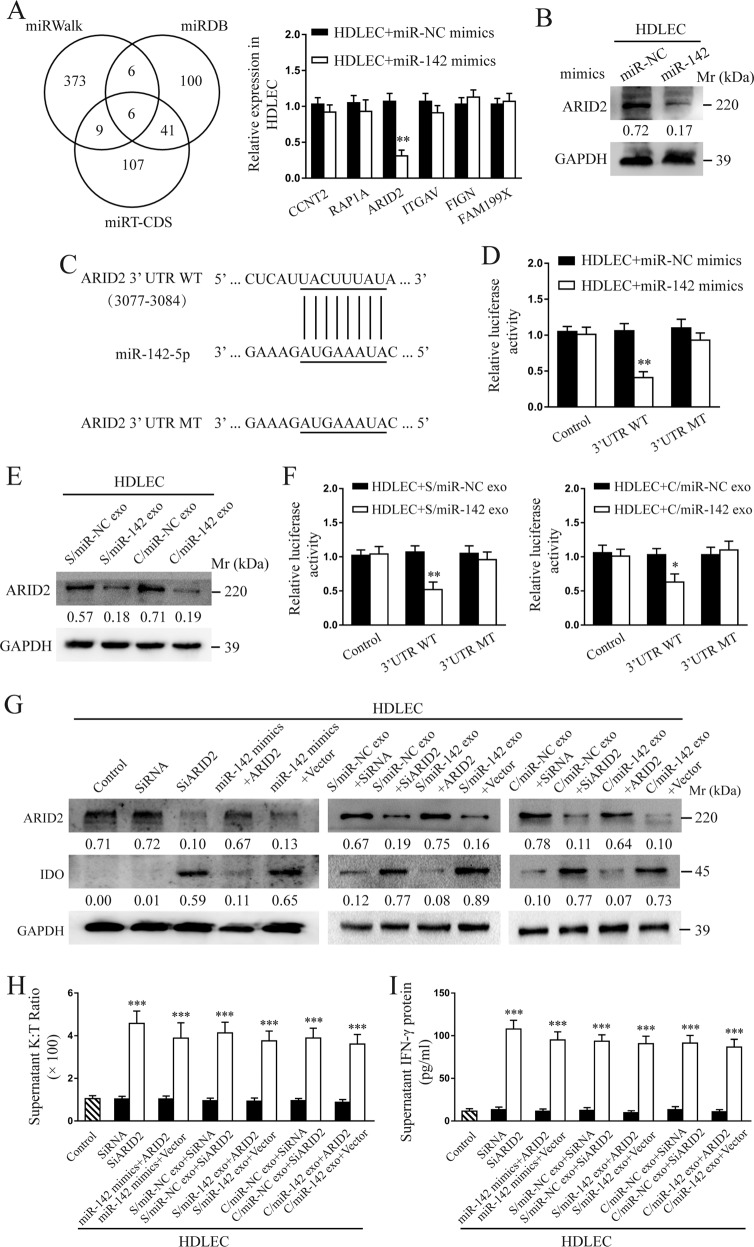
CSCC-secreted exosomal miR-142-5p directly targets ARID2 in HDLECs. **a** Overlap of three miRNA target bioinformatic prediction algorithms. The expression of *CCNT2*, *RAP1A*, *ARID2*, *ITGAV*, *FIGN*, and *FAM199X* in HDLECs pre-treated with miR-142-5p mimics or NC was detected by RT-qPCR. **b** The protein level of ARID2 in HDLECs pre-treated with miR-142-5p mimics or NC was analysed by western blotting. **c** The wild-type- and mutated-binding sites between miR-142-5p and ARID2 were shown. **d** Relative luciferase activity of HDLECs pre-treated with miR-142-5p mimics or NC was analysed. **e** The protein level of ARID2 in HDLECs pre-treated with indicated exosomes was analysed by western blotting. **f** Relative luciferase activity of HDLECs pre-treated with indicated exosomes was analysed. **g** The expression of ARID2 and IDO proteins in miR-142-5p-mimic/exosome-treated HDLECs transfected with plasmid-ARID2 or vector control and SiARID2- or SiRNA-treated HDLECs was detected by western blotting. **h** The K:T ratio of HDLEC supernatant after the indicated treatment was calculated. **i** IFN-γ protein level in the supernatant of HDLECs following indicated treatment was detected by ELISA. miR-142, miR-142-5p. S/miR-142 Siha/miR-142-5p, C/miR-NC Caski/miR-NC, C/miR-142 Caski/miR-142-5p, exo exosomes, WT wild-type, MT mutant. Error bars represent the mean ± SD of three independent experiments. *P < 0.05; **P < 0.01; ***P < 0.001.

Three bioinformatic tools (miRWalk, miRDB, and miRT-CDS) were employed to identify the candidate targets of miR-142-5p. All three programs predicted *CCNT2*, *RAP1A*, *ARID2*, *ITGAV*, *FIGN*, and *FAM199X* as candidate targets of miR-142-5p. To verify these predictions, we performed RT-qPCR analyses and found that only *ARID2* expression was significantly down-regulated in HDLECs following pre-treatment with miR-142-5p mimics (Fig. 4a). We also confirmed the decrease in the protein level of ARID2 in HDLECs treated with miR-142-5p mimics or exosomes containing miR-142-5p (Fig. 4b). We then determined the alignment between miR-142-5p and full-length ARID2 sequences and found an ARID2-coding sequence as a potential target of miR-142-5p (Fig. 4c). The wild-type and mutated miR-142-5p-binding sites were cloned into luciferase vectors and the luciferase activity was found to be significantly decreased in HDLECs co-transfected with the vector carrying the wild-type-binding site in the presence of miR-142-5p mimics, but not in HDLECs carrying the mutated-binding site (Fig. 4d). The protein level of ARID2 and luciferase activity also dramatically decreased in HDLECs treated with exosomes containing miR-142-5p (Fig. 4e, f). In addition, ARID2 expression level was examined in 116 human CSCC tissue microarrays using IHC and found to be significantly lower in advanced-stage (FIGO 2018, stages III and IV, *n* = 23) CSCC patients than in early-stage CSCC patients (FIGO 2018, stages I and II, *n* = 93) (Supplementary Fig. S4a). Further, ARID2 expression negatively correlated with miR-142-5p level in human CSCC tissues (Supplementary Fig. S4b). Taken together, these results suggest that ARID2 is a direct target of miR-142-5p.

We transfected cells with a plasmid-overexpressing ARID2 without 3′-untranslated region (UTR) or a specific small-interfering RNA (siRNA) SiARID2 that suppressed the expression of ARID2. Western blot analysis confirmed that plasmid-ARID2 and SiARID2 effectively overexpressed and knocked down ARID2 expression in HDLECs, respectively (Fig. 4g). Further in vitro studies confirmed that ARID2 overexpression neutralised the ability of miR-142-5p mimics or exosomes containing miR-142-5p to induce IDO activity (Fig. 4g, h). In contrast, ARID2 knockdown replicated the effects of miR-142-5p overexpression in HDLECs by inducing functional IDO expression (Fig. 4g, h). As IFN-γ induces IDO expression [26, 27], we next assessed whether IFN-γ expression was induced by the miR-142-5p-ARID2 axis. miR-142-5p overexpression or ARID2 knockdown significantly up-regulated the lymphatic level of IFN-γ, whereas ARID2 overexpression decreased the up-regulated level of IFN-γ induced by miR-142-5p mimics or exosomes containing miR-142-5p (Fig. 4i).

### ARID2 recruits DNMT1 to the IFN-γ promoter and enhances its methylation

**Fig. 5:**
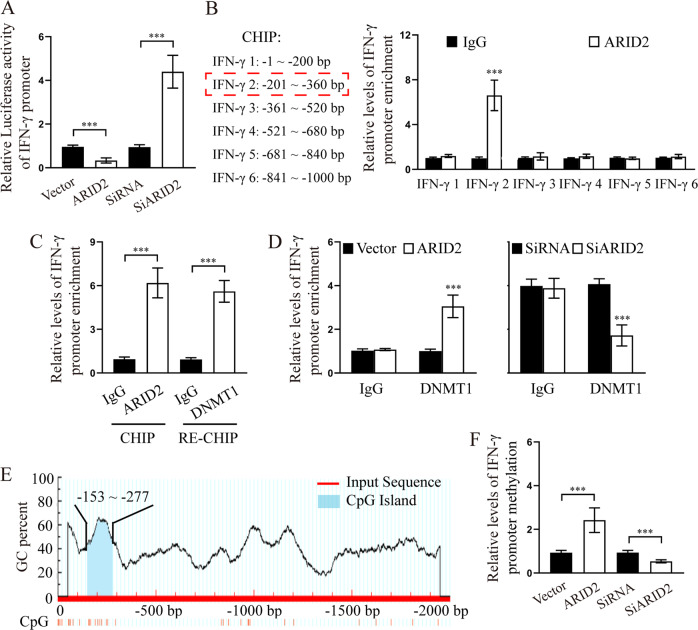
ARID2 enhances the methylation of IFN-γ promoter by recruiting DNMT1. **a** Relative luciferase activity of IFN-γ promoter in HDLECs following ARID2 overexpression or knockdown. **b** ChIP assay for the enrichment of ARID2 in the promoter region of IFN-γ in HDLECs. **c** ChIP-Re-ChIP assay to determine ARID2-DNMT1 co-occupancy in the promoter region of IFN-γ. **d** ChIP assay for the enrichment of DNMT1 in the promoter region of IFN-γ in HDLECs following ARID2 overexpression or knockdown. **e** Schematic map of the CpG island region in the IFN-γ promoter. f MSP assay to detect the methylation level of the IFN-γ promoter in HDLECs following ARID2 overexpression or knockdown. Error bars represent the mean ± SD of three independent experiments. ***P < 0.001.

As ARID2 mediated the expression of IFN-γ, we explored whether there exists a direct link between ARID2 and IFN-γ. As shown in Fig. 5a, luciferase reporter assay revealed the increase and decrease in IFN-γ promoter-driven luciferase activity in ARID2-silenced and ARID2-overexpressing cells, respectively. To investigate the role of ARID2 in IFN-γ expression, a ChIP-PCR assay was performed to determine whether the IFN-γ promoter region (from −1 to −1000 bp upstream of the exon) was occupied by ARID2. We found only one binding site (−201 to −360 bp) that showed strong interaction with ARID2 in HDLECs (Fig. 5b). Thus, ARID2 directly suppresses the transcription of IFN-γ.

As a subunit of the polybromo-associated BAF (PBAF) complex, ARID2 potentially interacts with epigenetic regulators and modulates gene transcription [28]. A recent study reported that DNMT1 was recruited as an epigenetic modulator by ARID2 to enhance the methylation of Snail promoter [29]. This observation promoted us to investigate the involvement of the ARID2-DNMT1 complex in the regulation of IFN-γ transcription. ChIP-RE-CHIP assay result suggests that DNMT1 and ARID2 co-occupied the same promoter region of IFN-γ (Fig. 5c). Further, the binding of DNMT1 to the IFN-γ promoter increased in ARID2-overexpressing HDLECs and decreased in ARID2-silenced cells, suggesting that ARID2 recruited DNMT1 to the IFN-γ promoter (Fig. 5d). We also found that the CpG island (−153 to −277 bp) overlapped with the binding site of the ARID2-DNMT1 complex at the IFN-γ promoter (−201 to −360 bp) (Fig. 5e). MSP assay result showed the up-regulation and down-regulation in IFN-γ promoter methylation in ARID2-overexpressing and ARID2-silenced HDLECs, respectively, following DNMT1 binding (Fig. 5f). Taken together, our results show that ARID2 suppressed the transcription of IFN-γ by recruiting DNMT1 to IFN-γ promoter, thereby elevating promoter methylation in HDLECs.

### miR-142-5p-activated HDLECs induce IDO expression to exhaust CD8^+^ T cells

**Fig. 6:**
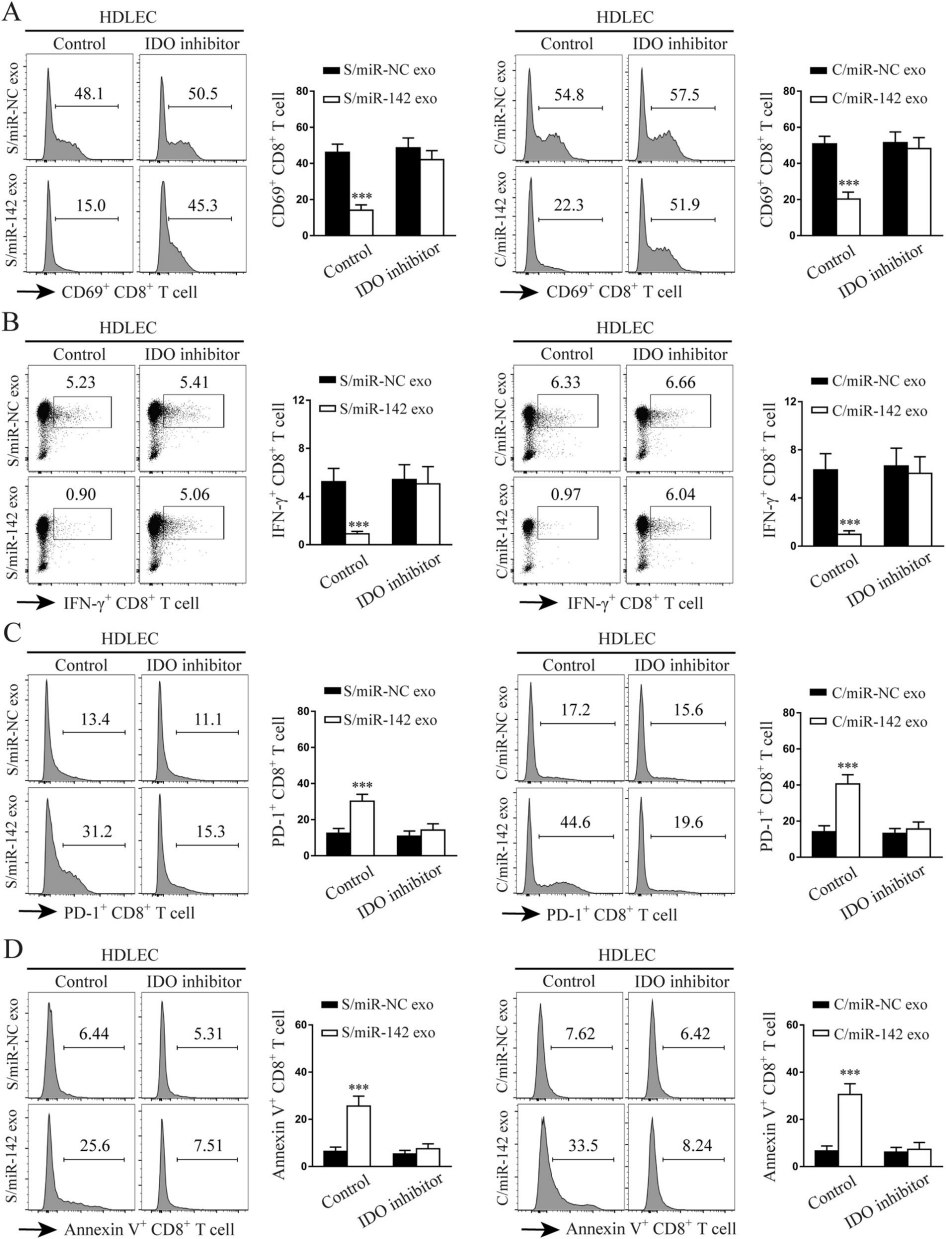
miR-142-5p-activated HDLECs induce IDO to exhaust CD8^+^ T cells. HDLECs pre-treated with indicated exosomes were cocultured with in vitro-activated CD8^+^ T cells at a ratio of 1:1 with or without 100 nM of an IDO inhibitor (BMS-986205). The expression of CD69 (**a**), IFN-γ (**b**), PD-1 (**c**), and annexin V (**d**) in CD8^+^ T cells was determined by FACS. S/miR-NC exo, Siha/miR-NC exosomes. S/miR-142 exo, Siha/miR-142-5p exosomes. C/miR-NC exo, Caski/miR-NC exosomes. C/miR-142 exo, Caski/miR-142-5p exosomes. Error bars represent the mean ± SD of three independent experiments. ***P < 0.001.

IDO promotes immune suppression through the attenuation of CD8^+^ T cell response [30]. To test whether HDLECs exposed to miR-142-5p contribute to suppressive effects on CD8^+^ T cell immunity, HDLECs pre-treated with exosomes with miR-142-5p or miR-142-5p mimics were co-cultured with in vitro-activated CD8^+^ T cells in the presence of a semipermeable transwell membrane. The CD8^+^ T cells were collected for flow cytometric analysis after culture. HDLECs pre-treated with exosomes containing miR-142-5p showed lower percentages of CD69^+^ (activated) and IFN-γ^+^ CD8^+^ (effector) T cells compared to the control cells (Fig. 6a, b) and had higher percentages of PD-1^+^ (inhibitory) and annexin V^+^ (apoptotic) CD8^+^ T cells (Fig. 6c, d). The blockade of IDO or miR-142-5p in HDLECs with an IDO inhibitor (BMS-986205) or miR-142-5p inhibitor (anti-142-5p) treatment, respectively, reversed the suppressive effects on CD8^+^ T cell functions induced by HDLECs pre-treated with exosomes containing high levels of miR-142-5p (Fig. 6a–d; Supplementary Fig. S5a–d). Likewise, HDLECs treated with miR-142-5p mimics also showed a decrease in the percentages of CD69^+^ and IFN-γ^+^ CD8^+^ T cells along with an increase in the percentages of PD-1^+^ and annexin V^+^ CD8^+^ T cells; these phenomena were reversed after IDO blockade (Supplementary Fig. S6a–d). Thus, IDO contributes to the immune suppression mediated by miR-142-5p-exposed HDLECs.

### Serum exosomal miR-142-5p levels correlate with systemic IDO activity and clinicopathological progression in patients with CSCC

**Fig. 7:**
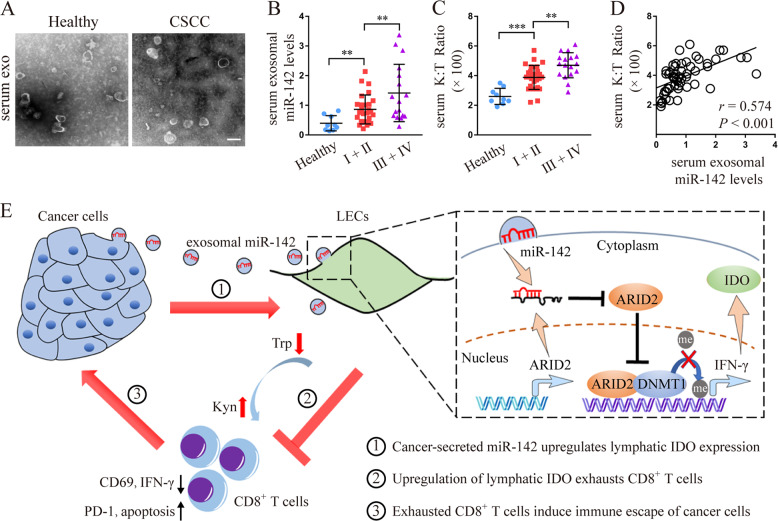
Serum exosomal miR-142-5p level correlates with systemic IDO activity and clinicopathological progression in patients with CSCC. **a** Morphology of exosomes isolated and purified from the serum of CSCC patients and healthy controls. Scale Bar: 50 nm. **b**–**c** miR-142-5p level and K:T ratio were detected in the serum exosomes of early-stage (FIGO 2018, stages I and II, *n* = 27) and advanced-stage (FIGO 2018, stages III and IV, *n* = 17) CSCC patients as well as healthy controls (*n* = 8). **d** Correlation analysis of exosomal miR-142-5p and K:T ratio in the serum of CSCC patients and healthy controls. **e** Proposed schematic diagram of exosomal miR-142-5p promoting lymphatic IDO expression to facilitate the immune escape of cancer cells via CD8^+^ T cell exhaustion. LECs, lymphatic endothelial cells. miR-142, miR-142-5p. Kyn, kynurenine. Trp, tryptophan. Error bars represent the mean ± SD of three independent experiments. **P < 0.01; ***P < 0.001.

To investigate whether the cancer-secreted miR-142-5p could be detected in the serum of CSCC patients, the exosomes were isolated from the serum of early-stage (FIGO 2018, stages I and II, *n* = 27) and advanced-stage (FIGO 2018, stages III and IV, *n* = 17) CSCC patients as well as healthy controls (*n* = 8) and characterised. The typical morphology and size range of the exosomes purified from the serum were consistent with those of the exosomes derived from cell culture supernatants (Fig. 7a). We then examined exosomal miR-142-5p from these serum samples. As shown in Fig. 7b, the serum exosomal miR-142-5p levels were higher in CSCC patients than in healthy controls. miR-142-5p levels were higher in the serum exosomes from advanced-stage CSCC patients than in those from early-stage CSSC patients. The serum K:T ratio showed a similar trend as serum exosomal miR-142-5p (Fig. 7c). The relationship between exosomal miR-142-5p and K:T ratio in the serum of CSCC patients was further investigated. As shown in Fig. 6d, exosomal miR-142-5p level positively associated with the K:T ratio in the serum of CSCC patients (*r* = 0.603, *P* < 0.001), indicating the potential value of serum exosomal miR-142-5p for the evaluation of IDO activity during CSCC progression.

In summary, our results show that CSCC-secreted exosomal miR-142-5p down-regulates the lymphatic expression of ARID2, inhibits DNMT1 recruitment to the IFN-γ promoter, and enhances IFN-γ transcription by suppressing promoter methylation, leading to elevated IDO activity to mediate CD8^+^ T cell exhaustion (Fig. 7e).

## Discussion

Multiple studies have demonstrated the clinical relevance of tumour-associated LECs and their involvement in malignant progression [31–33]. While tumour-associated LECs support the migration of tumour cells, they can enhance Foxp3-regulatory T cell functions and suppress responses of effector T cells, as demonstrated from the gain of a phenotype similar to that of lymphatic node LECs in mice [6, 34]. Our clinical data suggest that tumour-associated LVs helped to modulate anti-tumour immunity and their levels correlated with poor clinical outcome in CSCC patients. Thus, tumour-associated LVs are an integral component of the immunosuppressive TME. However, the mechanism underlying their function in mediating anti-tumour immunity is poorly understood.

In a recent study on recurrent or metastatic CSCC, the objective response rate was only 14.3% after treatment with anti-PD-1 antibodies for the blockade of immune checkpoints despite the selection of patients based on high PD-L1 expression score [35]. These outcomes suggest that additional immune checkpoint(s) mechanisms may control anti-tumour immunity. Extensive evidence suggests that IDO is associated with immune checkpoint(s) in many cancers [36–38], and our findings reveal the promotion of CD8^+^ T cell exhaustion via IDO mediated by CSCC-remodelled LECs. Although a previous study reported the anti-tumour activity of IDO^+^ LECs in vitro [39], the molecular mechanism leading to lymphatic IDO overexpression in the TME was not identified.

The immune TME is a dynamic system involving a complex intercellular crosstalk that develops and maintains an immune regulatory microenvironment to support tumour growth [40]. Exchange of cellular materials between cells through various paracrine and endocrine mechanisms is an important mean of intercellular crosstalk that may be executed by exosomes [25]. miRNAs from cancer-secreted exosomes are highly versatile regulators of this communication process [41]. In the present study, we found that high miR-142-5p expression correlated with peri-tumoural IDO^+^ LVs during CSCC progression in humans. The role of miR-142-5p as a tumour suppressor or oncogene seems to be cancer type-dependent [11, 12, 42]. Thus, a cancer-specific mechanism underlying the role of miR-142-5p may exist. Our observation that miR-142-5p was directly transferred from tumour cells to LECs via exosomes and up-regulated lymphatic IDO expression supported our initial correlative clinical data. In a previous study, we showed that CSCC-secreted exosomal miRNAs served as intercellular regulators between cancer cells and LECs and contributed to the remodelling of peri-tumoural LVs for metastatic spread [43]. Furthermore, high miR-142-5p expression in serum exosomes was associated with elevated IDO activity in advanced cancer stages. A recent study by Ferns et al. supported our idea that IDO activity correlated with poor clinicopathological parameters in CSCC [44]. Our results showed that aside from IDO-mediated anti-tumour immune activity, CSCC malignant progression altered the level of miR-142-5p in serum exosomes, indicating that serum exosomal miR-142-5p may discriminate between indolent and aggressive CSCC and contribute to the development of personalised diagnostic strategies for patients with different progression risks. We found that an IDO inhibitor abrogated the ability of LECs treated with exosomal miR-142-5p to suppress CD8^+^ T cells. In comparison with an anti-PD-1 treatment alone, the treatment with IDO inhibitor and anti-PD-1 agent increased ORR in CSCC [45]. Thus, therapies targeting miR-142-5p in combination with existing immune therapies such as IDO and PD-1 inhibitors may serve as comprehensive therapeutic approaches for CSCC patients at high risk of progression.

Numerous genes as direct targets of miR-142-5p have been identified. For instance, miR-142-5p directly suppresses the expression of suppressor of cytokine signalling 1 (SOCS1) to control the programming of profibrogenic macrophages via the activation of the signal transducer and activator of transcription (STAT) signalling [46]. Here, we identified a novel target ARID2 as the most down-regulated protein among the several predicted targets associated with miR-142-5p. ARID2 was proven to be a direct target of miR-142-5p by a luciferase reporter assay. In addition, we found that high ARID2 expression correlated with low miR-142-5p levels in human CSCC progression. Members of the ARID family have the ability to regulate transcription and are involved in cell differentiation and proliferation [47]. ARID2 functions as a subunit of the polybromo- and BRG1-associated factor or PBAF (SWI/SNF-B) chromatin remodelling complex, which facilitates ligand-dependent transcriptional activation by nuclear receptors [48]. Several studies have revealed the role of ARID2 as a significant tumour suppressor in many cancer subtypes [49]. Our data suggest that ARID2 protein level can be reduced in LECs by miR-142-5p mimics or exosomes containing miR-142-5p. Moreover, overexpression or knockdown of ARID2 could block or replicate the effects induced by miR-142-5p, respectively. IFN-γ-activated LECs can directly produce IDO in vitro [39], prompting us to determine whether IFN-γ could act as a downstream effector of the miR-142-5p-ARID2 axis. We found that high levels of miR-142-5p down-regulated ARID2 expression, leading to the up-regulation in the expression of IDO mediated by increased IFN-γ secretion.

Chromatin remodelling molecules often regulate gene expression by cooperating with epigenetic modulators, such as the E2F4-RBL2-HDAC1-BRM complex involved in proline-rich acidic protein 1 (PRAP1) transcriptional repression [50]. A recent study confirmed the recruitment of DNMT1 as an epigenetic modulator by ARID2 to enhance the methylation of gene promoter [29] that led us to investigate whether the ARID2-DNMT1 complex was also involved in the regulation of IFN-γ transcription. We verified the co-occupancy of ARID2 and DNMT1 at the same region of the IFN-γ promoter. In addition, the binding of DNMT1 to the IFN-γ promoter resulted in an increase in DNA methylation and was regulated by ARID2 expression. These data suggest that DNMT1 was recruited to the IFN-γ promoter by ARID2 to enhance promoter methylation.

In conclusion, our data provide evidence for the association of high levels of circulating exosomal miR-142-5p with increased IDO activity in advanced CSCC. The horizontal transfer of CSCC-secreted exosomal miR-142-5p to LECs results in the down-regulation of ARID2 expression, inhibition of DNMT1 recruitment to the IFN-γ promoter, and enhancement of IFN-γ transcription via the suppression of promoter demethylation, eventually elevating IDO activity for CD8^+^ T cell exhaustion. The newly identified miR-142-5p–ARID2–DNMT1–IFN-γ–IDO axis in LECs appears to be a critical molecular mechanism underlying CSCC progression and may serve as a novel diagnostic and therapeutic target for CSCC patients at high risk of progression.

## Supplementary information

Supplementary Figure S1

Supplementary Figure S2

Supplementary Figure S3

Supplementary Figure S4

Supplementary Figure S5

Supplementary Figure S6

Supplementary Figure Legends

Supplementary materials and methods

Supplementary tables
