# Supplementary figures and images for: Exosome-derived miR-142-5p remodels lymphatic vessels and induces IDO to promote immune privilege in the tumour microenvironment
Source: Cell Death Differ. 2020 Sep 14;28(2):715–29. doi: 10.1038/s41418-020-00618-6 (PMC7862304; doi:10.1038/s41418-020-00618-6)

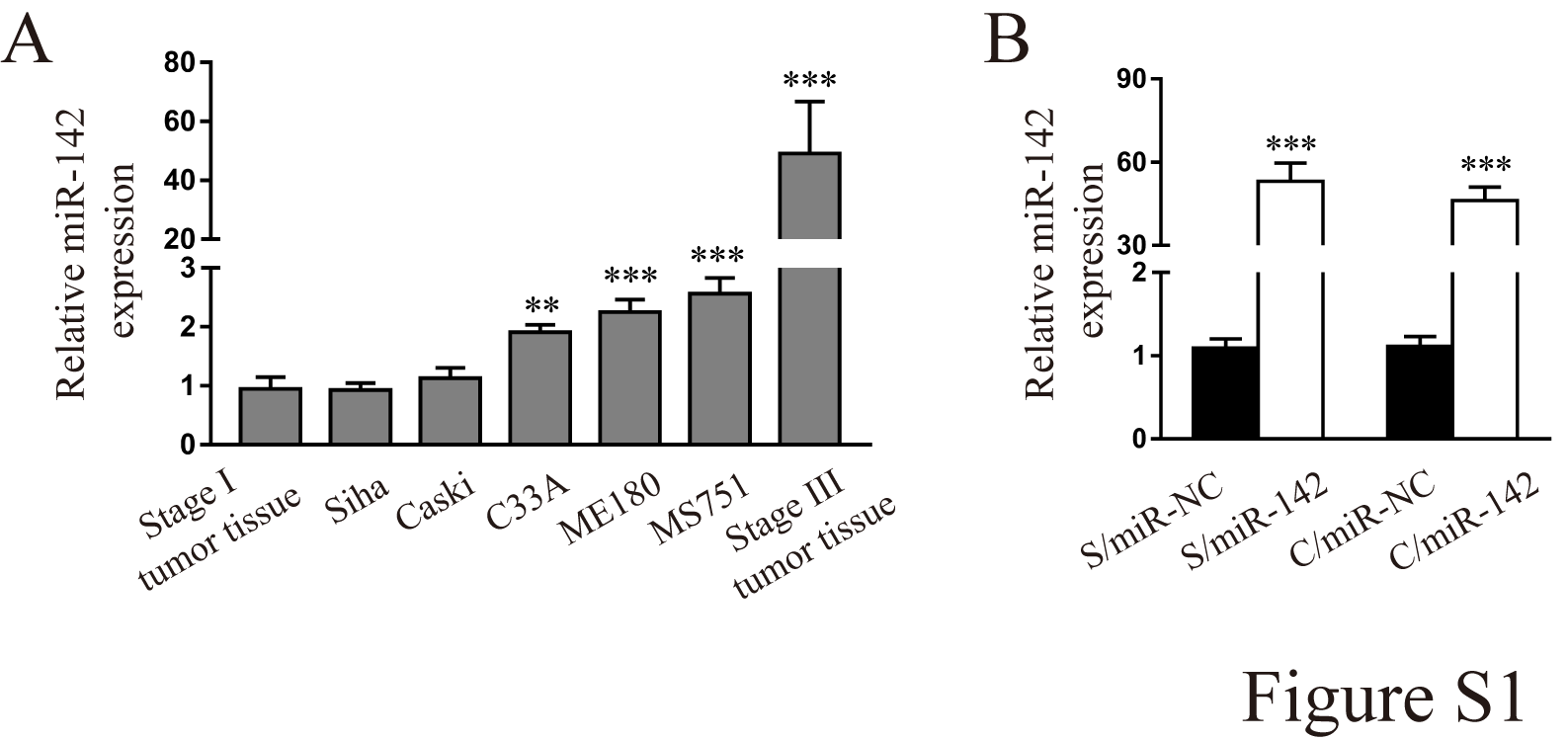

Supplement: Supplementary file 1 — Supplementary Figure S1 [file 41418_2020_618_MOESM1_ESM.tif]

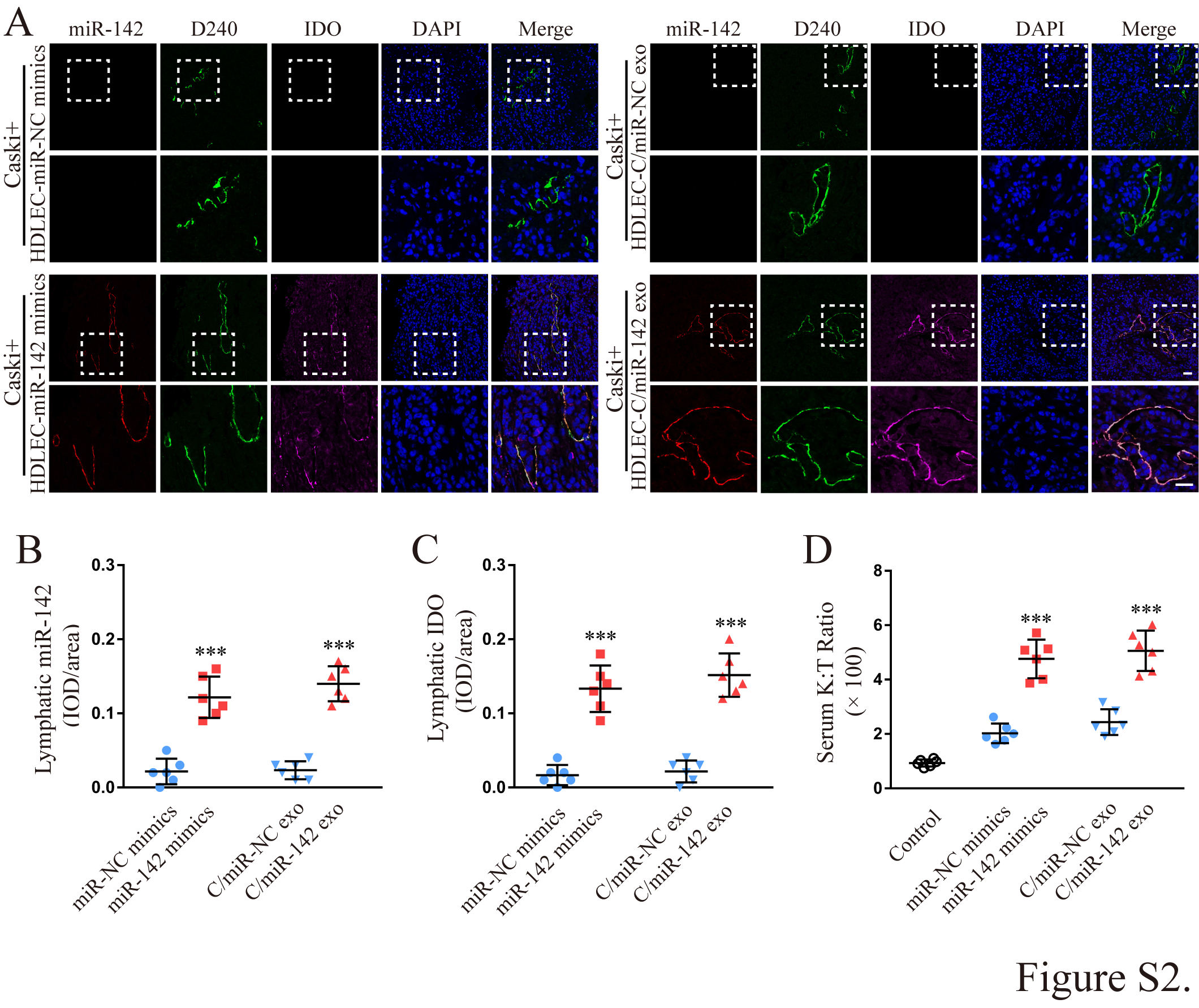

Supplement: Supplementary file 2 — Supplementary Figure S2 [file 41418_2020_618_MOESM2_ESM.tif]

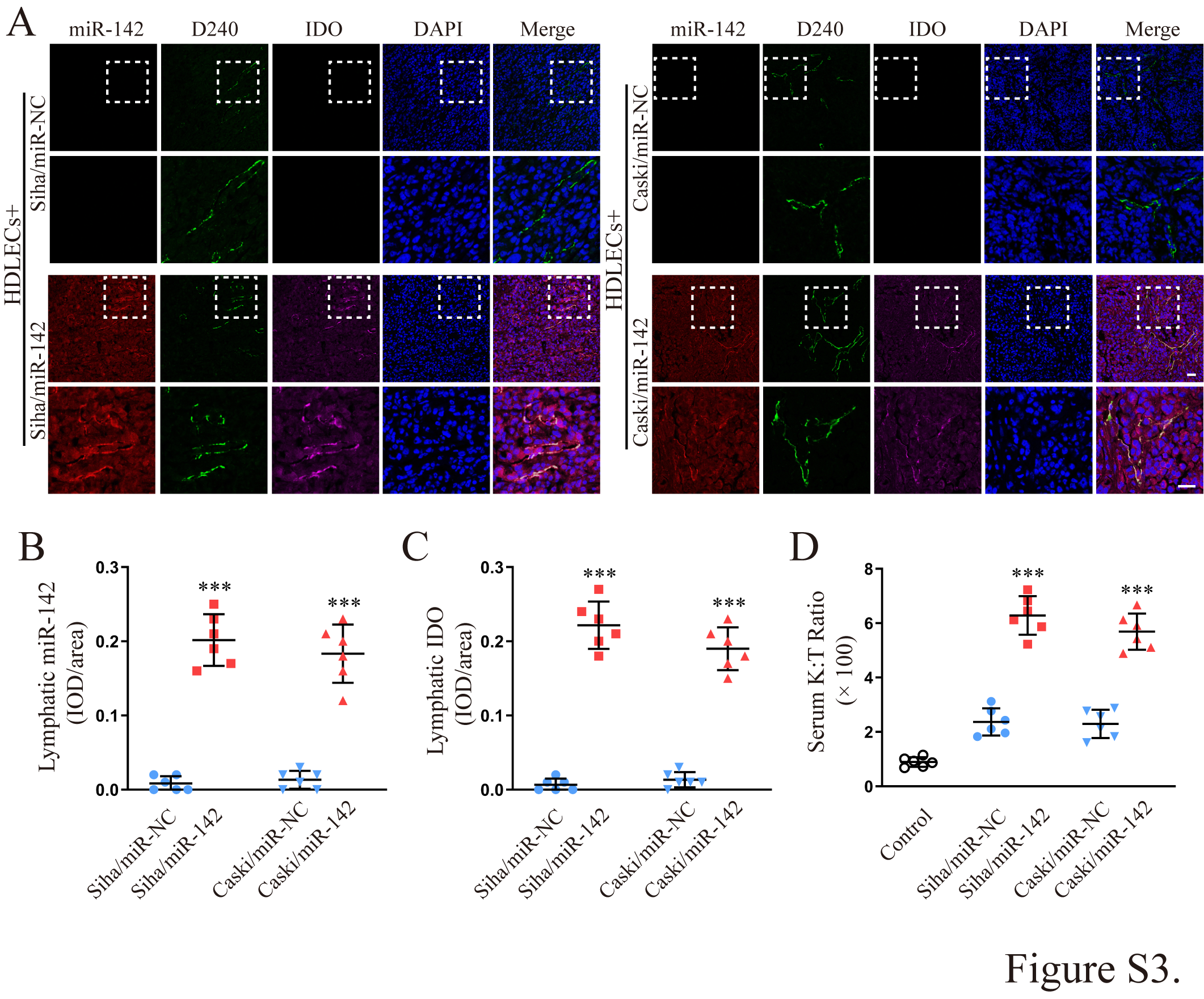

Supplement: Supplementary file 3 — Supplementary Figure S3 [file 41418_2020_618_MOESM3_ESM.tif]

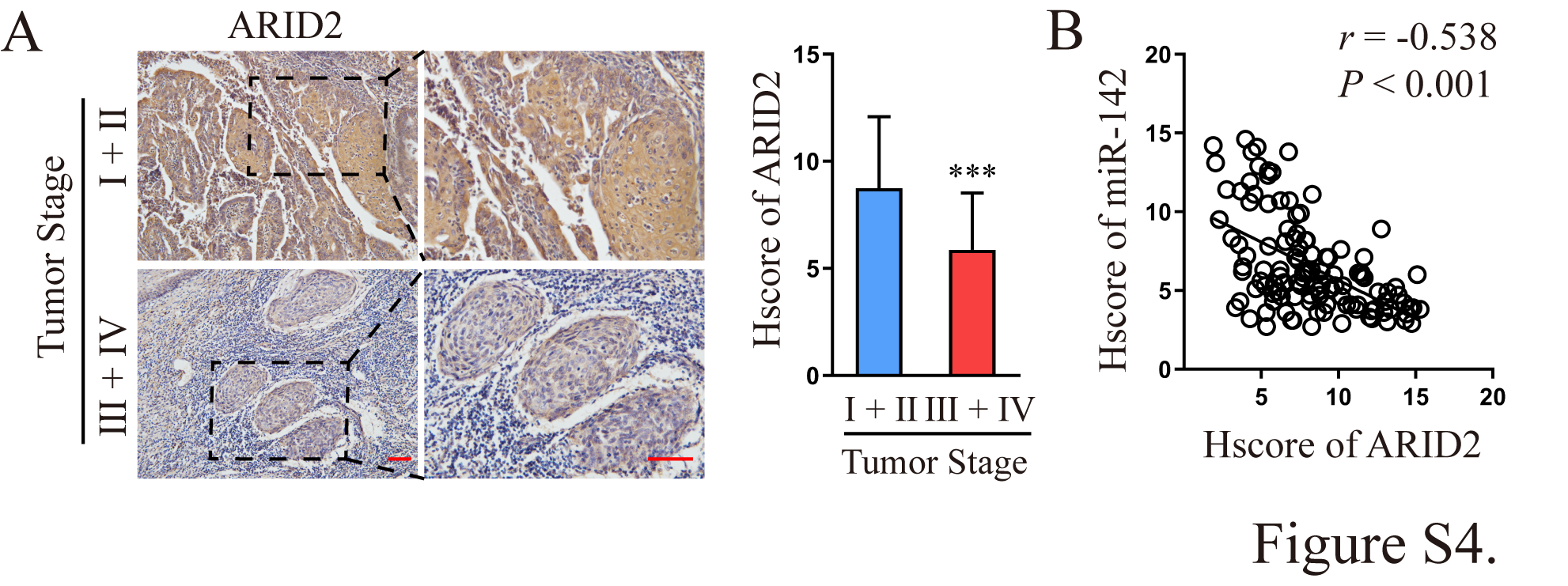

Supplement: Supplementary file 4 — Supplementary Figure S4 [file 41418_2020_618_MOESM4_ESM.tif]

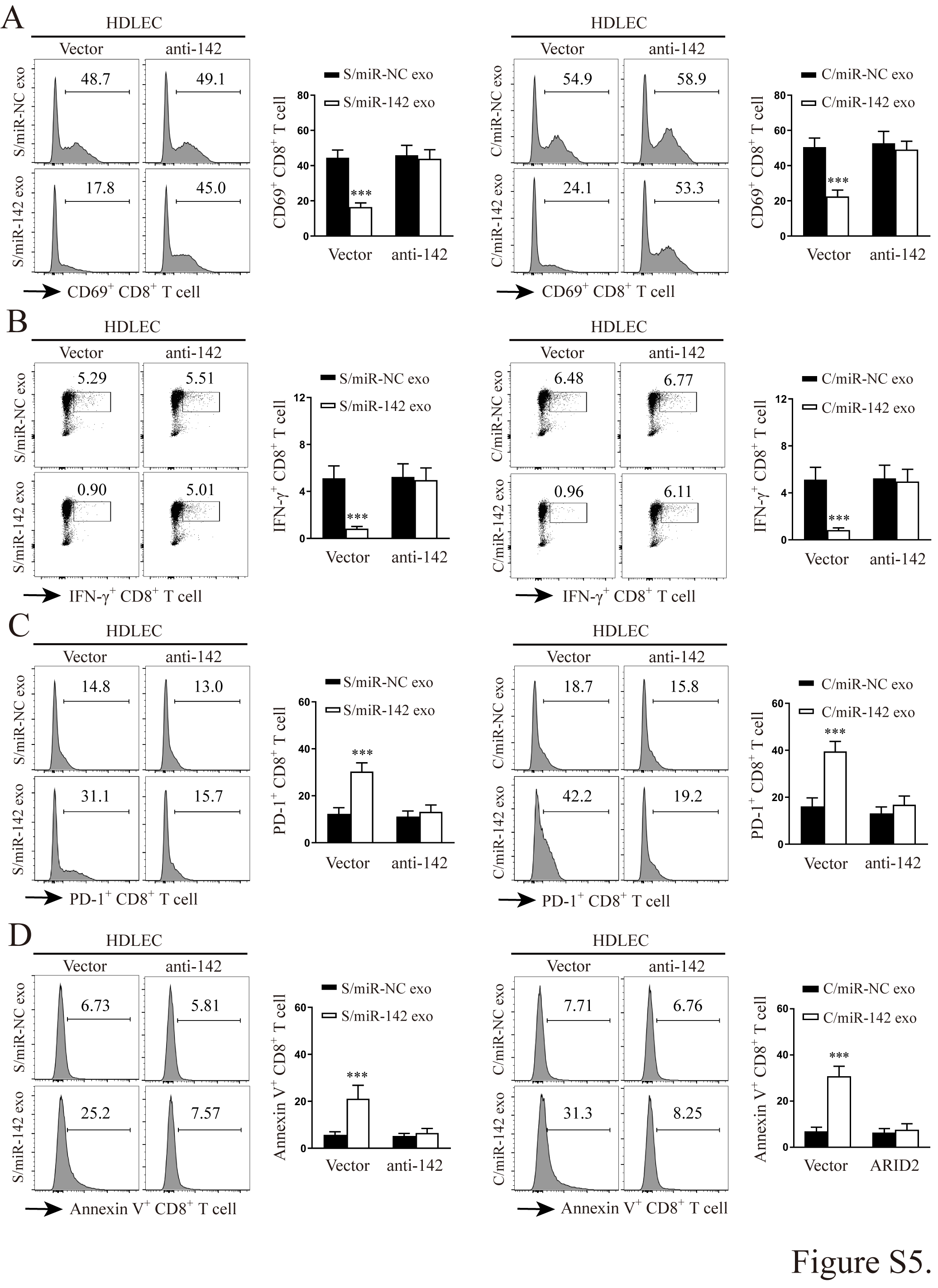

Supplement: Supplementary file 5 — Supplementary Figure S5 [file 41418_2020_618_MOESM5_ESM.tif]

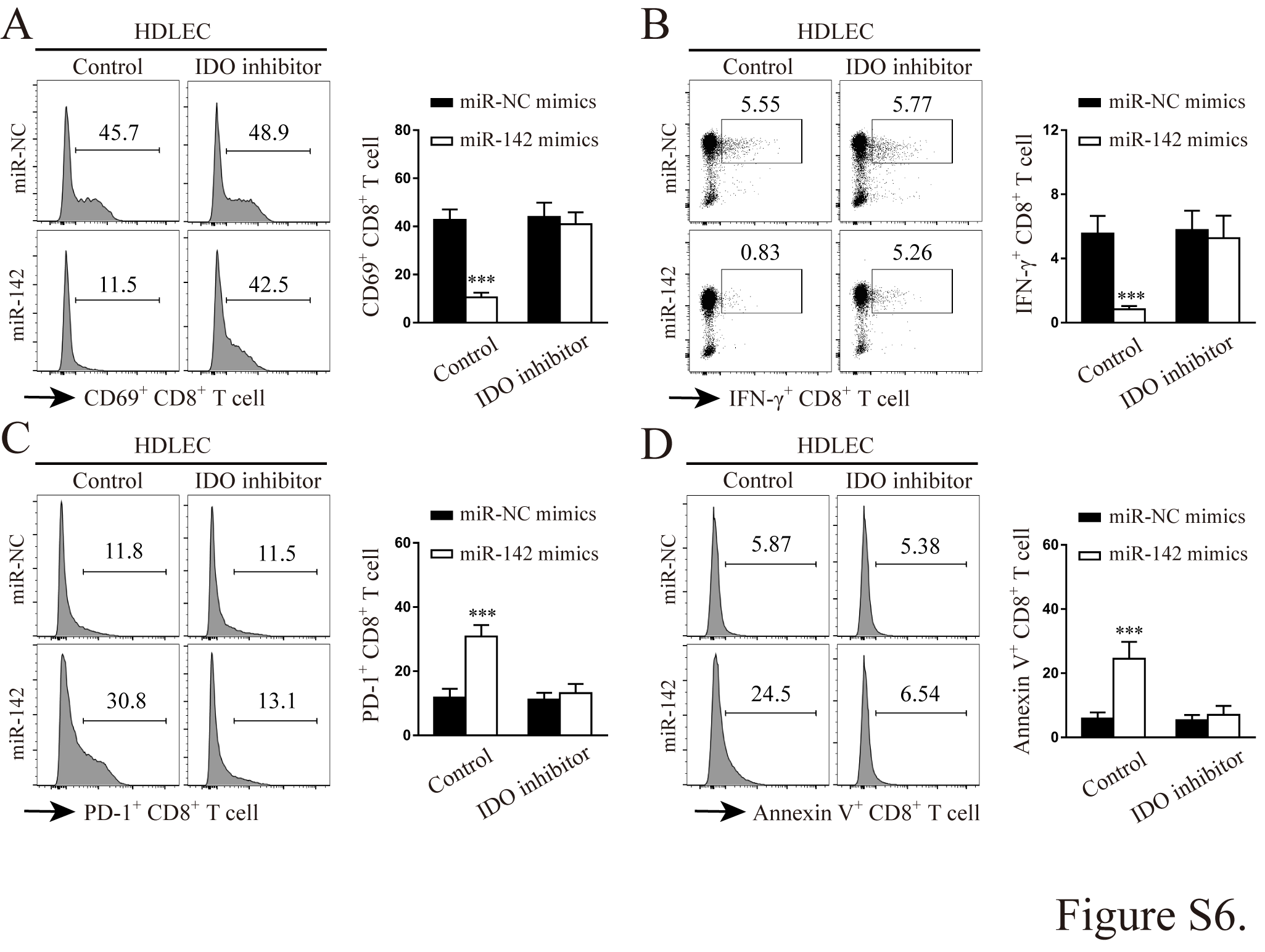

Supplement: Supplementary file 6 — Supplementary Figure S6 [file 41418_2020_618_MOESM6_ESM.tif]
