# Supplementary material for: Exosome-derived miR-142-5p remodels lymphatic vessels and induces IDO to promote immune privilege in the tumour microenvironment
Source: Cell Death Differ. 2020 Sep 14;28(2):715–29. doi: 10.1038/s41418-020-00618-6 (PMC7862304; doi:10.1038/s41418-020-00618-6)
Supplement: Supplementary file 7 — Supplementary Figure Legends [file 41418_2020_618_MOESM7_ESM.docx]

**Fig S1.** miR-142-5p can be transferred to HDLECs via CSCC-secreted exosomes, related to Fig. 2. a miR-142-5p levels in tumor tissues and CSCC cell lines were detected by qRT-PCR. b miR-142-5p levels in Siha and Caski transfected with lentivectors overexpressing miR-142-5p or NC were detected by qRT-PCR. S/miR-NC, Siha/miR-NC. S/miR-142, Siha/miR-142-5p. C/miR-NC, Caski/miR-NC. C/miR-142, Caski/miR-142-5p. Error bars represent the mean ± SD of three independent experiments. **, P<0.01; ***, P<0.001.

**Fig S2.** HDLECs pre-treated with Caski-secreted exosomal miR-142-5p upregulates its IDO expression *in vivo*, related to Fig. 3. a Representative micrographs of miR-142-5p, IDO and D240 immunofluorescence staining in sections of xenograft tumors are shown. Scale Bar: 50 µm. b-d Lymphatic expression of miR-142-5 and IDO and serum kynurenine to tryptophan (K:T) ratio in each group was calculated. C/miR-NC, Caski/miR-NC. C/miR-142, Caski/miR-142-5p. exo, exosomes. Error bars represent the mean ± SD of three independent experiments. ***, P<0.001.

**Fig S3.** Untreated HDLECs mixed with Siha/miR-142-5p or Caski/miR-142-5p upregulates lymphatic IDO expression *in vivo*, related to Fig. 3. a Representative micrographs of miR-142-5p, IDO and D240 immunofluorescence staining in sections of xenograft tumors are shown. Scale Bar: 50 µm. b-d Lymphatic expression of miR-142-5 and IDO and serum kynurenine to tryptophan (K:T) ratio in each group was calculated. Error bars represent the mean ± SD of three independent experiments. ***, P<0.001.

**Fig S4.** ARID2 expression correlates negatively with miR-142-5p expression and CSCC progression, related to Fig. 4. a ARID2 expression was detected in serial sections between the early stages (FIGO 2018, stages Ⅰ and Ⅱ, *n* = 93) and advanced stages (FIGO 2018, stages Ⅲ and Ⅳ, *n* = 23) of CSCC. Scale Bar: 50 µm. b Correlation between ARID2 and miR-142-5p expression in human CSCC tissue was analyzed. miR-142, miR-142-5p. Error bars represent the mean ± SD of three independent experiments. ***, P<0.001.

**Fig S5.** HDLECs incubated with miR-142-5p inhibitors (anti-142-5p) reversed the suppressive effects on CD8^+^ T cell function induced by HDLECs pre-treated with exosomes containing high levels of miR-142-5p, related to Fig 5**.** HDLECs pre-treated with indicated exosomes were un-contracted cocultured with in vitro-activated CD8^+^ T cells at a ratio of 1:1 in the presence of miR-142-5p inhibitor or Control. The expression of CD69 (a), IFN-γ (b), PD-1 (c) and Annexin V (d) on CD8^+^ T cells were determined by FACS. S/miR-NC exo, Siha/miR-NC exosomes. S/miR-142 exo, Siha/miR-142-5p exosomes. C/miR-NC exo, Caski/miR-NC exosomes. C/miR-142 exo, Caski/miR-142-5p exosomes. anti-142, anti-142-5p. Error bars represent the mean ± SD of three independent experiments. ***, P<0.001.

**Fig S6.** HDLECs incubated with IDO inhibitor reversed the suppressive effects on CD8^+^ T cell function induced by HDLECs pre-treated with miR-142-5p mimics, related to Fig 5. HDLECs pre-treated with miR-142-5p mimics or NC were un-contracted cocultured with *in vitro*-activated CD8+ T cells at a ratio of 1:1 in the presence of 100 nM IDO inhibitor (BMS-986205) or Control. The expression of CD69 (a), IFN-γ (b), PD-1 (c) and Annexin V (d) on CD8^+^ T cells were determined by FACS. miR-142, miR-142-5p. Error bars represent the mean ± SD of three independent experiments. ***, P<0.001.
