# Supplementary material for: Exosome-derived miR-142-5p remodels lymphatic vessels and induces IDO to promote immune privilege in the tumour microenvironment
Source: Cell Death Differ. 2020 Sep 14;28(2):715–29. doi: 10.1038/s41418-020-00618-6 (PMC7862304; doi:10.1038/s41418-020-00618-6)
Supplement: Supplementary file 8 — Supplementary materials and methods [file 41418_2020_618_MOESM8_ESM.docx]

**Supplementary materials and methods**

**Staining assessment**

For semi-quantitative evaluation of IDO and miR-142-5p expression in tissue sections, German Immuno-Reactive Score was applied as previously described. The Hscore was calculated by combining the proportion of positively stained cells and the intensity of staining. The staining intensity was rated on a scale of 1 to 4, with 1 representing no staining, 2 weak staining, 3 moderate staining, and 4 strong staining. No staining was scored as 0; staining of 1% to 10% as 1; 11% to 50% as 2; 51% to 80% as 3; and 81% to 100% as 4. The raw data were converted to Hscore by multiplying the quantity score and the intensity score. Human specimens with a Hscore ≥ 4 were defined as high expression; specimens with a Hscore < 4 were defined as low expression. The ratios of IDO^+^ LVs to total LVs ≥ 15% were classified as high IDO^+^ LVs; The ratios of IDO^+^ LVs to total LVs < 15% were classified as low IDO^+^ LVs.

**Luciferase activity assay**

For analysis of miR-142-5p targeting ARID2, the putative miR-142-5p complementary site in the 3’-UTR of ARID2 or its mutant sequence was cloned into the pmiR-RB-REPORT vector (RiboBio Inc.). Then, pmiR-RB-REPORT-ARID2-3’UTR-WT or pmiR-RB-REPORT- ARID2-3’UTR-MT and the control vector pmiR-RB-REPORT vector were co-transfected into HLDECs with miR-142-5p mimics or indicated exosomes. For analysis of ARID2 targeting IFN-γ, wild-type IFN-γ promoter plasmids containing firefly luciferase reporters were co-transfected with an internal control pRL-TK containing a full length Renilla luciferase gene (Promega) into HDLECs with ARID2 overexpression or knockdown following the manufacturer’s protocol. After 48 h transfection, cells were analyzed by using a Dual-Luciferase Reporter Assay System (Promega). Firefly luciferase activity was normalized to Renilla luciferase activity for each transfected well. All assays were performed in triplicate and each experiment was repeated three times.

**ELISA assay**

IFN-γ protein levels was detected in the culture supernatant of HDLECs after indicated treatment using a human IFN-γ ELISA Kit (Thermo Fisher) according to the manufacturer’s protocol.
