# Supplementary material for: Exosome-derived miR-142-5p remodels lymphatic vessels and induces IDO to promote immune privilege in the tumour microenvironment
Source: Cell Death Differ. 2020 Sep 14;28(2):715–29. doi: 10.1038/s41418-020-00618-6 (PMC7862304; doi:10.1038/s41418-020-00618-6)
Supplement: Supplementary file 9 — Supplementary tables [file 41418_2020_618_MOESM9_ESM.docx]

**Supplementary Tables**

**Table S1. Detailed primer sequences in the study**

|  | Forward | Reverse |
| --- | --- | --- |
| CCNT2 | TGCTTACCTTCAACAGACTCAAG | GGGTGTTCAATGGTGATCTCAAA |
| RAP1A | CGTGAGTACAAGCTAGTGGTCC | CCAGGATTTCGAGCATACACTG |
| ARID2 | AAGGACAGCGGGTACTTCAGA | CCAAGAGCTTAACATTGCCCT |
| ITGAV | GCTGTCGGAGATTTCAATGGT | TCTGCTCGCCAGTAAAATTGT |
| FIGN | GTAGCACCAGTGTTTATGGCT | GTAGGCTTCAACTTTGTGGGC |
| FAM199X | CACACCAACAGGTGGAACCTA | TGACCAATCCCAGTAAGTGCTA |
| GAPDH | CCATCAATGACCCCTTCATTGACC | GAAGGCCATGCCAGTGAGCTTCC |
| SiARID2 | GGAGAUGGUUCUCAUUUAATT | UUAAAUGAGAACCAUCUCCTT |
| SiRNA | UUCUCCGAACGUGUCACGUTT | ACGUGACACGUUCGGAGAATT |
| IFN-γ 1 | TCTGTCTCATCGTCAAAGGACCC | CACACCATTCAAGGACTGGAAATTTTT |
| IFN-γ 2 | CTATCATCCCTGCCTATCTGTCACCA | ACCAGAAAGCAAGGAAAGAATGCG |
| IFN-γ 3 | CCAGTCCTTGAATGGTGTGAAGT | GCTCTAGATCAGCACTGCCCA |
| IFN-γ 4 | AACCGCATTCTTTCCTTGCTTTCT | GTGTAGAGAATATTCCCAGGGGGAG |
| IFN-γ 5 | GAGAGGCCCTAGAATTTCGTTTTTCAC | GCATGGTGGTGTATGCCTGTAAT |
| IFN-γ 6 | GAGCCACTGCGTCTGGAAC | GGGTGGATCACAAGGTCAGGAG |
| M primer | TTTTGATTAATATAGTGAAATTTCGT | TCACCCAAACTAAAATACAATAACG |
| UM primer | TTGATTAATATAGTGAAATTTTGT | CCCAAACTAAAATACAATAACACA |

**Table S2. The primary antibodies used in Western Blot**

| Name | Description | Company | Product Code |
| --- | --- | --- | --- |
| anti-CD9 | rabbit monoclonal | Abcam | ab92726 |
| anti-HSP70 | mouse monoclonal | Abcam | ab2787 |
| anti-TSG101 | rabbit monoclonal | Abcam | ab125011 |
| anti-IDO | rabbit monoclonal | Cell Signaling Technology | 86630 |
| anti-ARID2 | rabbit monoclonal | Cell Signaling Technology | 82342 |
| anti-GAPDH | rabbit monoclonal | Cell Signaling Technology | 2118 |
